# Special issue on enhancing vector refractoriness to trypanosome infection-foreword

**DOI:** 10.1186/s12866-018-1279-4

**Published:** 2018-11-23

**Authors:** Adly M. M. Abd-Alla, George Tsiamis, Drion G. Boucias

**Affiliations:** 1 Insect Pest Control Laboratory, Joint FAO/IAEA Division of Nuclear Techniques in Food & Agriculture, Vienna International Centre, P.O. Box 100, 1400 Vienna, Austria; 2 Department of Environmental and Natural Resources Management, University of Patras, 2 Seferi St, 30100 Agrinio, Greece; 3 Entomology and Nematology Department, University of Florida, 970 Natural Area Drive, Gainesville, FL 32611 USA

Sleeping sickness or Human African Trypanosomosis affects people in approximately 37 countries in sub-Saharan Africa and is caused by *Trypanosoma brucei gambiense* and *Trypanosoma brucei rhodesiense*. The *T. brucei gambiense* is responsible for about 95% of the chronic cases in central and western Africa, whereas *T. brucei rhodesiense* causes the acute form of the disease in eastern Africa. The parasites are transmitted by blood-feeding tsetse fly species belonging to the genus *Glossina*. Other *Glossina*-transmitted trypanosomes also infect cattle and cause a disease called nagana, a Zulu word meaning “to be depressed”. Nagana or African Animal Trypanosomosis results in millions of dollars of economic losses to countries that can ill afford such losses.

The management of nagana, based on the recurrent treatment of livestock with trypanocidal drugs is costly and not sustainable due to increased resistance of the parasites. In attempts to develop more sustainable approaches to the management of the disease in Africa, several governments adopted the sterile insect technique (SIT). This technique, when integrated with other suppression tactics, has been successful in eradicating a population of *Glossina austeni* on the Island of Unguja (Zanzibar), Republic of Tanzania and in almost eradicating a population of *Glossina palpalis gambiensis* in the Niayes region of Senegal. It relies on limiting the reproductive capacity of the tsetse flies by releasing large numbers of mass-reared sterile males.

So far, International Atomic Energy Agency (IAEA)-supported projects that have an SIT component for tsetse and trypanosomosis control focused on Animal African Trypanosomes causing nagana but future projects could target potential human disease transmission. In this context, it is imperative to take strict measures to ensure that released sterile tsetse flies cannot transmit the disease-causing trypanosome parasite. Therefore, development of tsetse fly strains refractory to trypanosome infection is highly desirable as a simple and effective method of ensuring vector incompetence of the released flies.

Coordinated Research Projects are the most effective tool to gather scientists from all over the world to work together on solving important problems in Agency Member States. The Sterile Insect Technique (SIT) is an environment friendly control tactic to manage insect pests using area-wide integrated pest management approaches. Five years ago, a coordinated research project (CRP) entitled “Enhancing Vector Refractoriness to Trypanosome Infection” was initiated under the auspices of the Joint Division of Nuclear Techniques in Food and Agriculture of the Food and Agriculture Organization (FAO) and the IAEA. Adly Abd-Alla of the FAO/IAEA coordinated the project included 23 scientists from 19 countries, representing a broad range of expertise, to gain a deeper knowledge of the tripartite interactions between the tsetse fly vectors, their symbionts, and trypanosome parasites and to acquire a better understanding of mechanisms that limit the development of trypanosome infections in tsetse and how these may be enhanced. The studies involved detailed investigations into the biology of the insect in relationship to the causative trypanosomes, parasites, and symbionts, as well as epidemiological investigations of the disease in various parts of Africa. The scientists convened four times at about 18-month intervals to report their findings and to coordinate their research.

This special issue is comprised of the final research results of the CRP (19 research papers), together with an introductory review paper highlighting the objectives and the main achievements and three reviews to provide background to the project. Further research, carried out by CRP participants and collaborators during the CRP and published previously, is listed in Table 1 of Kariithi et al., [1].

It is our hope, as guest editors, that this issue of the BMC Microbiology will provide an extensive treatise on the tsetse flies and its symbionts, parasites, and pathogens that will inform the insect pathology and symbionts community about advances made in this extremely important problem that continues to affect both humans and cattle in sub-Saharan Africa.
